# A new method for identifying the cut ends in canalicular laceration

**DOI:** 10.1038/srep43325

**Published:** 2017-02-24

**Authors:** Wenyan Peng, Yandong Wang, Bowei Tan, Haiying Wang, Xuehua Liu, Xuanwei Liang

**Affiliations:** 1 State Key Laboratory of Ophthalmology, Zhongshan Ophthalmic Center, Sun Yat-sen University, Guangzhou, 510060, P. R. China; 2 Brookdale University Hospital and Medical Center, Brooklyn, NY 11212, USA

## Abstract

To locate the proximal and distal cut ends of the canaliculus following trauma is the most difficult part of canalicular repair, especially in patients with complex acute canalicular lacerations and late presenting canalicular lacerations. Previously, irrigation and air-injection technique are reported and widely used to locate the cut ends of lacerated canaliculus. However, we have developed a novel technique in which with a 23 Ga fiber optic light pipe is used to identify the cut ends of the canaliculus allowing silicone tube intubation of the lacrimal system. The mean time from initiation of the identification of the cut ends of the canaliculus to insertion of the silicone tube was 5 minutes. In this study, the cut ends were successfully identified by using this novel method in 33 cases of acute and late presenting canalicular laceration for canalicular reconstruction without any complications. This light-guided technique may represent an improvement in the surgical repair of canalicular lacerations.

Canalicular laceration is a very common trauma to the lacrimal system^1^. Finding the canalicular cut ends is the first key step during the operation of canalicular reconstruction. A number of techniques including irrigating air, water, colored or viscous agents through the intact canaliculus have been reported to aid in the identification of the canalicular cut ends for repairing canalicular lacerations^2^,^3^,^4^. In the clinical practice, we find that any of these methods are defined as difficult procedures or very restricted in patients with a narrow canaliculus, persistent wound bleeding, and especially canalicular scarring in late presenting canalicular laceration. Here, we describe a new method in which a 23 Ga fiber optic light pipe is used to identify the cut ends in complex acute canalicular laceration and late presenting canalicular laceration.

## Methods

### Ethics Statement

This study was approved by the institutional review board (IRB) of ZhongShan Ophthalmic Center, Sun Yat-sen University, Guangzhou, China. And the methods were implemented in accordance with the approved guidelines. Informed consent was obtained from all patients.

### Patients

We screened 40 patients who had canalicular lacerations and required surgical repairment in Zhongshan Ophthamic Center from January 2013 to June 2013. The inclusion criteria was patients with acute or late presenting canalicular lacerations, age 18–65, without diabetes mellitus, structural heart disease or coronary heart disease and other systemic diseases. We excluded patients with concurrent orbital fracture, nasal bone fracture, abnormal electrocardiogram, any signs of local infections or systemic infections, and uncompliant patients. The commitment to be compliant with follow up was the prerequisite to be included to this study. 33 patients were included to our study, informed consent and the commitment to follow up were signed by all subjects. In all 33 patients, 21 patients had acute canalicular lacerations (less than 1 week), and 12 patients had late presenting canalicular lacerations (more than 1 week). All the surgeries were performed by the same surgeon (Dr. Xuanwei Liang) under local anesthesia.

### Surgical technique

**Figure 1:**
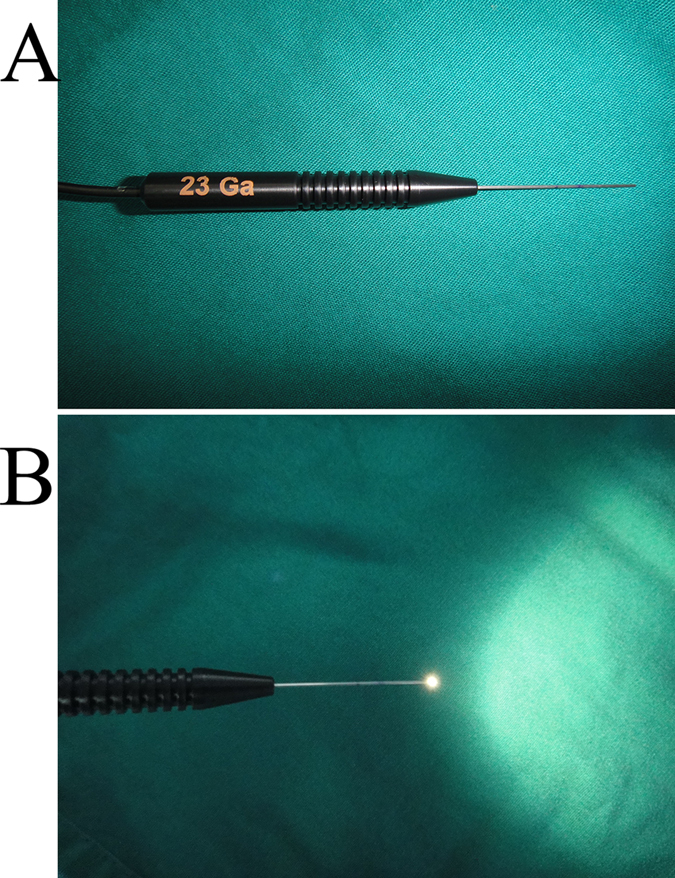
(**A**) Photograph of the fiber optic light pipe, the tip edge is polished to avoid secondary injury. (**B**) Photograph of the fiber optic light pipe with the light up.

A fiber optic light pipe (model 23 G, 0.6 mm in diameter, Alcon Laboratories Inc., Fort Worth, TX, USA) was used in this operation (Fig. 1A). We made a mark 10 mm distal to the tip of pipe (the distance from upper or lower lacrimal punctum to the lacrimal sac is approximately 10 mm), so that we could estimate the length of the distal ends of laceration to the lacrimal punctum through observing the position of the mark when the light pipe inserted into the upper or lower lacrimal punctum (Fig. 1B). The surface and edge of the fiber optic light pipe tip were polished to avoid potential chafing, cutting or forming a false passage. The fiber optic light pipe was autoclave sterilised prior to use.

**Figure 2:**
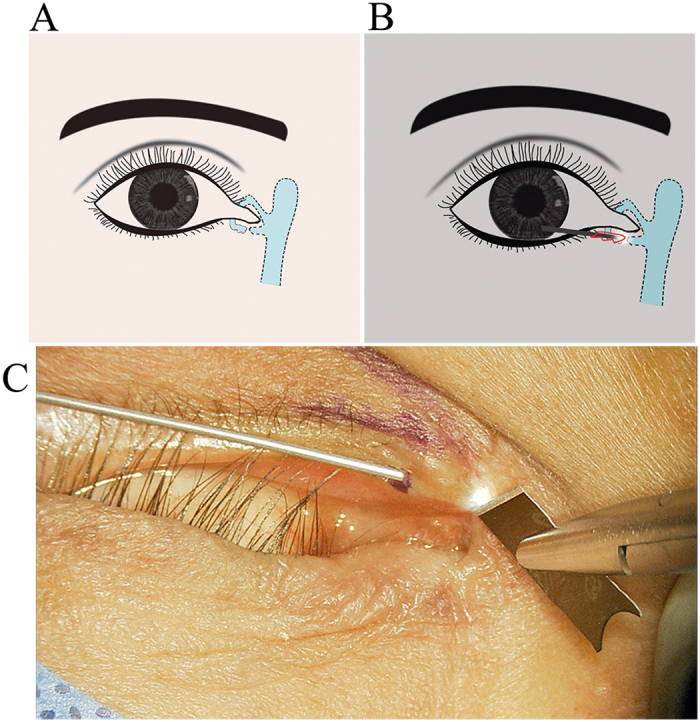
(**A**) Schematic diagram of an late presenting canalicular lacerations. (**B**) Schematic diagram of the light source go through the lacrimal canaliculus from the lacrimal punctum, which could provide an unobstructed view of light from the old injuried site, and then we could easily and precisely locate the distal laceration end and make an incision at the canalicular scarring. (**C**) Intraoperative clinical photograph: The light source illuminates a focussed cut end.

In late presenting lacerations, the ends of laceration was generally obscured by scar tissues, the most important part for surgical repair is to maximally preserve the remaining normal canalicular tissue and accurately identify the position of the laceration ends, therefore the crux is to determine where to start the cut. Our method using the 23 G model fiber optic light pipe inserted from the upper or lower lacrimal punctum, and we cut when the light pipe met the resistance (the location of the lacerated canalicular), then we could easily identify distal ends of laceration (Fig. 2A,B and C), usually proximal ends of laceration could be found easily when we cut off the scar tissue little by little under the microscope.

**Figure 3:**
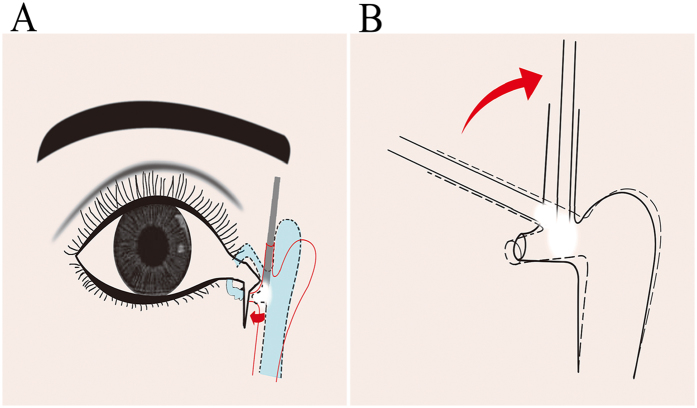
(**A**) Schematic diagram of locating the cut end of an acute or late presenting lacerated canaliculus with our white light source through the 23 Ga fiber optic light pipe. The fiber optic light pipe was passed through the upper canaliculus. The surgeon then dimmed the microscope light to enable visualization of the light source in the lacrimal sac from the proximal lacerated port. (**B**) Schematic diagram of the fiber optic light pipe was rotated to place the tip closely adjacent to the proximal end of the lacerated canaliculus. The cut end was identified at the light brightness.

In acute lacerations which usually we could identify the fresh lacerations distal ends easily but had difficulties to locate the proximal ends of laceration especially when distance of lacerated distal cut end and the lacrimal punctum exceeded 7 mm or had concurrent edema or hemorrhage. We could insert the light pipe into the lacrimal passage through either the upper or lower (the opposite of injury site) intact punctum after being dilated and ended at the lacrimal sac then turn the light pipe 90 degrees to be vertical to lacrimal passage. Concurrently, an assistant helped expose lacerated parts of lacrimal canaliculus. The surgeon then dimmed the microscope light to enable visualization of the light source in the lacrimal sac from the proximal lacerated port (Fig. 3A and B), so that we could easily and precisely locate the proximal end of lacerations and make an incision when necessary. This method could also be well applied in repairing late presenting canalicular lacerations when we were unsure where were proximal ends of lacerations.

**Figure 4:**
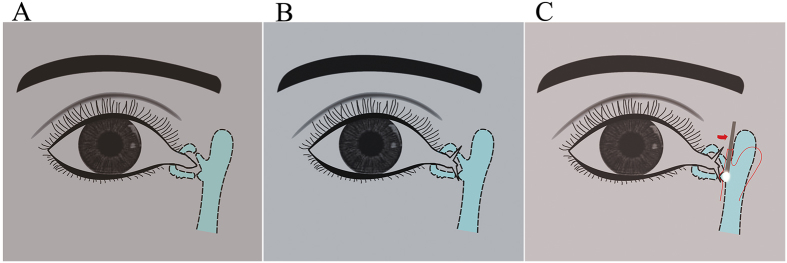
(**A**) Schematic diagram of an ipsilateral bicanalicular lacerations. (**B**) Schematic diagram of an identified distal ends in ipsilateral bicanalicular lacerations by the methods illustrated in Fig. 3. (**C**) Schematic diagram of the light pipe inserted from the identified proximal end of the laceration (either upper or lower) to the lacrimal sac, identify the proximal ends of laceration by the translucent light source from the lacrimal sac.

In cases of ipsilateral bicanalicular lacerations (acute or late presenting), by the methods illustrated in Fig. 2, we could easily identify distal ends of laceration. Usually one of either upper or lower proximal ends of laceration could be found with ease when we were cutting the scar tissue. However, when the distal end of laceration is too distal (more than 7 mm) from the canalicular punctum, we also could insert the light pipe from the identified proximal end of the laceration (either upper or lower) to the lacrimal sac, so that we could identify the proximal ends of laceration by the translucent light source from the lacrimal sac (Fig. 4A,B and C).

Later we inserted a silicone tube and gently guided towards the proximal and distal ends. The lacerated pericanalicular tissue and medial canthal tendon were repaired using 5-0 and 6-0 vicryl sutures, respectively. The skin wound was closed with a marginal lid-laceration repairing method by using 6-0 silk, as described in previous study^5^. Tobramycin Dexamethasone Eye Drops (S.A.ALCON-COUVREUR N.V. Belgium) or 0.5% Levofloxacin Eye Drops (Santen (China), pharmaceutical, co). and 0.1% fluorometholone eye drops (Santen (China), pharmaceutical, co) were used postoperatively for 2–4 weeks.

The improvement of symptoms, tearing, anatomic reposition and functional recovery of canaculus were evaluated at each follow up visit. We took out the silicone tube at least 3 months after surgery, if patient had concurrent ipsilateral bicanalicular laceration, we deferred to 6 months post-operatively, and the irrigation was performed right after we took out the tube. Anatomic reposition was achieved when the silicone tube was successfully placed.

## Results

We screened all 40 patients who had canalicular lacerations and 33 patients were enrolled according to our including criteria, 7 patients were excluded according to our excluding criteria. There were 3 upper, 16 lower, and 2 ipsilateral bicanalicular among the 21 patients with acute canalicular lacerations, and 7 lower and 5 ipsilateral bicanalicular among the 12 patients with late presenting canalicular lacerations. All 33 cases were actively followed up in outpatient clinic during a 6-month period. No subject declined to participate and no case showed signs of localized or systemic infections post-operatively. We found that anatomic reposition of the canalicular laceration were achieved in all 33 cases (100%). We confirmed that re-canalizations were also achieved in all the cases after irrigation of the lacrimal passage during follow-up visits. No complications caused by this method of identifying the cut ends such as iatrogenic damage and restenosis were observed during the follow-up period.

## Discussion

Many techniques have been utilized in the standard practice for identifying the cut ends of a lacerated canaliculus, such as the air bubble test, injection of water, colored or viscous agents liquid. However, these tests have their drawbacks. The air bubble test requires a closed system, and the colored solutions may always make the operative field obscure^2^. Nevertheless, although various methods have been reported, many surgeons continue to encounter difficulties in finding the laceration ends.

In this study, we found that using white light from a 23 Ga model fiber optic light pipe to identify the laceration ends produced less iatrogenic trauma, and was proved to be a simple and practical technique. Typically, the closer the laceration is to the lacrimal sac, the more difficult it is to locate the cut ends of the lacerated canaliculus.

In China, as a developing country, we have many patients with complex canaliculus lacerations secondary to indirect or blunt injuries with eyelid avulsion, such as blunt trauma to the lateral face with a closed fist, falls and motor vehicle accidents, multiple forces acting in several directions to create the avulsion with or without other soft tissue injuries. Identification of the cut ends of the canaliculus is difficult and time consuming when there is concurrent local soft tissue edema or hemorrhage, especially when distance of lacerated distal cut end and the lacrimal punctum exceeds 7 mm.

However, the white light penetrates soft tissue easily, no matter how close the laceration is to the lacrimal sac. Our technique was proved to be simple, safe, and effective. In addition, this method can be used to identify the cut end not only in solitary lacrimal canaliculus lacerations but also in dual lacerations, localizing in both upper and lower lacrimal canaliculi. In cases of ipsilateral bicanalicular lacerations, we are able to identify one side of the ipsilateral lacrimal canaliculus first, no matter upper or lower, then proceed to the other laceration. However, the efficiency of this technique in concurrent nasal bone fracture requires further evaluation.

## Additional Information

**How to cite this article:** Peng, W. *et al*. A new method for identifying the cut ends in canalicular laceration. *Sci. Rep.* **7**, 43325; doi: 10.1038/srep43325 (2017).

**Publisher's note:** Springer Nature remains neutral with regard to jurisdictional claims in published maps and institutional affiliations.
